# Larval size in acanthocephalan parasites: Influence of intraspecific competition and effects on intermediate host behavioural changes

**DOI:** 10.1186/1756-3305-5-166

**Published:** 2012-08-09

**Authors:** Lucile Dianne, Loïc Bollache, Clément Lagrue, Nathalie Franceschi, Thierry Rigaud

**Affiliations:** 1 Equipe Ecologie Evolutive, UMR CNRS 6282 Biogéosciences, Université de Bourgogne, 6 Boulevard Gabriel, 21000, Dijon, France; 2 Present address: Department of Zoology, University of Otago, P.O. Box 56, Dunedin, 9054, New Zealand

**Keywords:** *Pomphorhynchus laevis*, *Gammarus pulex*, Intraspecific competition, Parasite larval size, Host behavioural manipulation, Phototaxis

## Abstract

**Background:**

Parasites often face a trade-off between exploitation of host resources and transmission probabilities to the next host. In helminths, larval growth, a major component of adult parasite fitness, is linked to exploitation of intermediate host resources and is influenced by the presence of co-infecting conspecifics. In manipulative parasites, larval growth strategy could also interact with their ability to alter intermediate host phenotype and influence parasite transmission.

**Methods:**

We used experimental infections of *Gammarus pulex* by *Pomphorhynchus laevis* (Acanthocephala), to investigate larval size effects on host behavioural manipulation among different parasite sibships and various degrees of intra-host competition.

**Results:**

Intra-host competition reduced mean *P. laevis* cystacanth size, but the largest cystacanth within a host always reached the same size. Therefore, all co-infecting parasites did not equally suffer from intraspecific competition. Under no intra-host competition (1 parasite per host), larval size was positively correlated with host phototaxis. At higher infection intensities, this relationship disappeared, possibly because of strong competition for host resources, and thus larval growth, and limited manipulative abilities of co-infecting larval acanthocephalans.

**Conclusions:**

Our study indicates that behavioural manipulation is a condition-dependant phenomenon that needs the integration of parasite-related variables to be fully understood.

## Background

Parasitic organisms have to exploit hosts to optimize growth and/or transmission while coping with the limited amount of host resources. Trade-offs between damaging the host when exploiting its resources and benefits taken from this exploitation is general in parasites. Macro-parasite fitness is often linked to intra-host growth, resulting in intraspecific variations in individual body size. Parasites with complex life-cycles exploit one or several intermediate hosts before reaching the definitive host where they mature and reproduce. Consequently, larger larval sizes in intermediate hosts are associated with fitness benefits in definitive hosts like increased establishment success and survival [1-3], or larger adult body size and higher fecundity [4]. However, larval stages of such parasites face trade-offs between high growth rate and prudent exploitation of intermediate hosts. Directional selection towards large larval stages should thus be stabilized by the necessity to maintain intermediate host viability until parasite transmission [5]. Parasite size is not only modulated by host characteristics and evolutionary trade-offs, it is also strongly influenced by the presence and number of other parasites sharing the same host [4,6-9]. Studies on intra- and interspecific competition between macro-parasites are common, both in intermediate and definitive hosts e.g. [4,8,10-18]. This intra-host competition generally induces a negative relationship between individual size and parasite load e.g. [2,4,8,9,16,19].

Many complex-life cycle parasites have developed the ability to alter the phenotype of their intermediate hosts – increasing transmission probabilities –, a phenomenon called parasitic manipulation [20-24]. Growth strategies could thus interact with manipulation abilities since both are assumed to be energetically costly [21]. Franceschi *et al.* showed that developmental rates (i.e. time to reach the infective stage) of acanthocephalan larvae traded-off with individual parasite manipulation ability [23]. Slow developing larvae eventually induced much higher manipulation intensities (i.e. phototaxis reversal) in intermediate hosts. Two studies on cestode parasites support the idea that larger larval size favours parasitic manipulation [24,25]. However, in these parasites, size is correlated with age. Whether apparent size effects on host manipulation were due to parasite age (as observed in an acanthocephalan; [26]) or if it was a direct size effect cannot be discriminated.

*Pomphorhynchus laevis* is a fish acanthocephalan using amphipod crustaceans as intermediate hosts. Several amphipod behavioural traits are altered by *P. laevis*[27-31]. Notably, phototaxis is reversed after the parasite reaches infective cystacanth stage [26]. Manipulation intensity is highly variable and this variability is still incompletely understood. Factors affecting behavioural manipulation intensity can be environmental (e.g. time of day and season, see respectively [30] and [23]) or “parasite-related” (e.g. cystacanth age and larval developmental rate; [23,26]). However, effects of final cystacanth size on behavioural manipulation have never been investigated. Origins of size variations in *P. laevis* cystacanths are also poorly documented although individual cystacanth size is influenced by inter- and intraspecific competition [8,32].

Using experimental infection of *G. pulex* by *P. laevis*, we investigated whether cystacanth size modulates the intensity of host behavioural alterations. Since some parasite life-history traits vary between parasite strains (i.e. genetic lineages; [23]), we evaluated size differences between parasite sibships and tested if these values influenced their ability to alter amphipods phototaxis. These tests were made under different degrees of intra-host competition (single infections vs. infections involving two or more than two parasites). To avoid any confounding effect of parasite age on size, all experimental infections were synchronous.

## Methods

### Host and parasite collection

*Gammarus pulex* were collected in a small tributary of the Suzon River (Burgundy, eastern France; 47°24’12.6”N, 4°52’58.2”E). This population is known to be free of *P. laevis*[26]. In the laboratory, gammarids were acclimatized for 4 weeks prior to infection experiments in 37×55×10 cm aquaria containing dechlorinated, UV-treated and aerated tap water. Temperature was maintained at 15 ± 1°C under a 12:12 hours light:dark cycle and elm (*Ulmus laevis*) leaves were supplied as food.

Naturally-infected chubs (*Leuciscus cephalus*) were captured by electrofishing in the Ouche River (47°17’44.92”N, 5°02’44.63”E). Fish were anaesthetized using a Eugenol solution (Sigma-Aldrich), killed by decapitation and dissected to collect adult parasites from their intestines. Eggs were obtained by dissecting female acanthocephalans and placed in 400 μL of water. Parasite tissues were preserved in 300 μL of 100 % ethanol for molecular identification since two closely-related species of acanthocephalan parasites, *Pomphorhynchus laevis* and *P. tereticollis*, co-occur in Burgundy [33]. This molecular identification was made following Franceschi *et al.*[26].

### Infection procedure

Maturity of parasite eggs from each clutch was assessed under a Nikon compound microscope. Egg clutches of eight female *P. laevis* coming from eight different fish were selected according to egg maturity for experimental infection. In this experiment, a clutch is considered as a “parasite sibship” [23].

Prior to infection, gammarids were deprived of food for 24 h. Controlled infections were then carried out as described in Franceschi *et al.*[26]. Only male gammarids were used to avoid potential confounding effects of female reproductive stage on infection outcomes. Two amphipods were placed in a 6 cm diameter dish filled with water. Parasite eggs were deposited on a 1 cm² dry elm leaf placed in the dish (100 eggs per gammarid). Amphipods were then allowed to feed for 48 h. For each treatment (one treatment corresponding to infection with one parasite sibship), 108 male gammarids were used. Uninfected leaves were provided to control groups. Amphipods were then rinsed, placed in 0.5 L aquaria and maintained under standard conditions (water at 15 ± 1°C, 12:12 hours light:dark cycle). Eighteen individuals from the same treatment (exposed to eggs from the same female parasite) were haphazardly assigned to each aquarium.

From the sixth week after infection, gammarids were inspected once a week under a binocular microscope to detect the presence of parasites. Individuals harbouring visible *P. laevis* larvae were isolated. These gammarids were then checked twice a week until parasites reached cystacanth stage (final larval stage infective to definitive hosts). Infected host reaction to light was measured a day after the parasite had reached cystacanth stage and again fifteen days later. These measures are thereafter referred as “early” and “late” phototaxis, respectively [26].

A single gammarid was introduced into a horizontal tube filled with aerated water and divided into a dark and a light zone of equal size. After 5 min of acclimatization, amphipod position was recorded every 30 s for 5 min; a score of 0 was given to individuals located in the dark area and a score of 1 was given to those in the lighted area. Summed scores ranged from 0 (always in the dark, strongly photophobic) to 10 (always in the light, strongly photophilic). Gammarids were then measured (body height at the level of the fourth coxal plate basis) using a Nikon SMZ 1500 stereoscopic microscope and Lucia G 4.81 software and dissected. Cystacanths were counted and measured (length and width). Cystacanth volume was then calculated as the volume of an ellipsoid: V = (πLW²)/6, with L and W being respectively length and width of the cystacanth [34].

### Statistics

All tests were performed using JMP 7.0 Software (SAS Institute Inc.) and were two-tailed. P-values < 0.05 were considered significant.

Two linear models were carried out to investigate the effects of parasite sibship (i.e. clutch) and infection intensity (i.e. number of cystacanths per host, divided into three categories: 1, 2 and more than 2 cystacanths, respectively) *(i)* on mean cystacanth volume within a host and *(ii)* on variation coefficient of cystacanth volume. Host size may also partly explain parasite size [e.g. 2, 8], and was therefore included in the models. A linear model was also used to analyse the effects of these same factors on the size of the larger cystacanth per host.

Phototaxis scores never met normality nor homoscedasticity conditions, even after transformations. Therefore, to allow analyses of these two factors and their interaction simultaneously, early and late phototaxis scores were transformed into two categorical variables (i.e. scores below and above median scores). These categories were analysed using a logistic regression model. Early phototaxis and late phototaxis were separated in each analysis.

In all statistical models, non significant interactions and factors were removed from the analyses.

## Results

### Parasite volume per host

**Figure 1:**
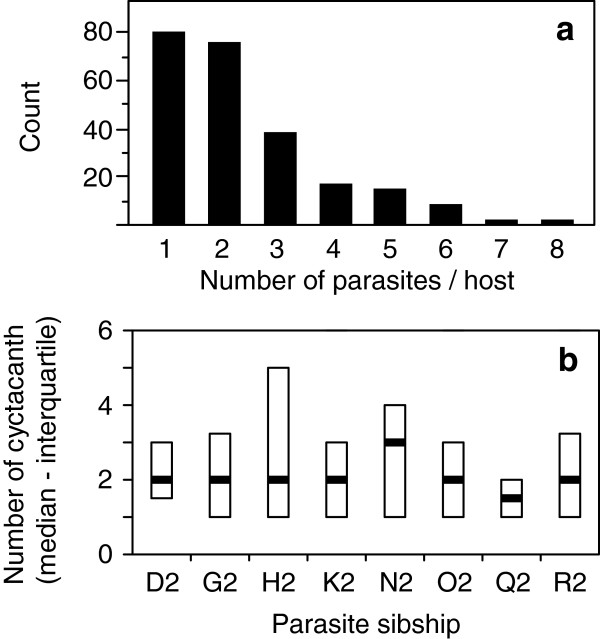
**Parasite intensity within a host and effect of parasite relatedness. (a)** Frequency distribution of the number of cystacanths per host. **(b)** Number of cystacanths per host according to parasite sibship.

Experimentally infected amphipods contained between 1 and 8 parasites (see distribution on Figure 1a). Infection intensity was not influenced by parasite sibship (Likelihood ratio: *χ²*_*7*_ = 10.82, *P* = 0.15; Figure 1b). Development of these parasites was almost synchronized (68.5 % of the 238 parasites reached the cystacanth stage 61 days post-exposure, the others one week later), and did not differ between sibship (Kruskal-Wallis: *χ²*_*7*_ = 6.54, *P* = 0.47).

**Figure 2:**
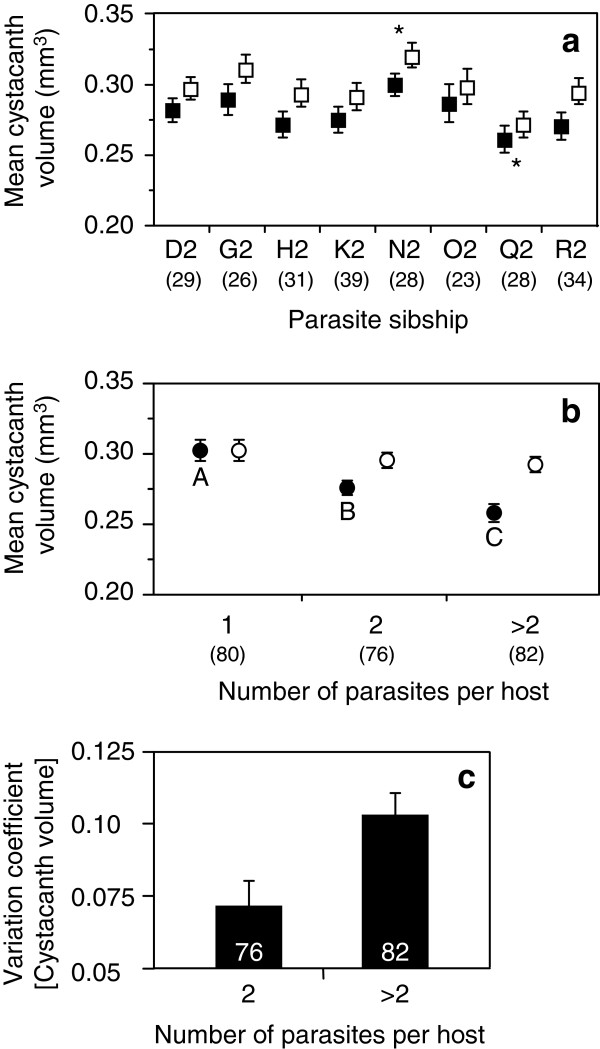
**Variability of mean and largest cystacanth volumes within a host. (a)** Effect of parasite sibship on mean cystacanth volume (black symbols) and volume of the largest cystacanth within a host (open symbols). Error bars represent standard errors. Stars indicate sibships where both mean and larger cystacanth volumes were either larger or smaller compared to other sibships (after Tuckey HSD post-hoc test). **(b)** Effect of infection intensity on mean cystacanth volume (black symbols) and volume of the largest cystacanth within a host (open symbols). Error bars represent standard errors. Symbols marked with different letters indicate significant differences among groups (after Tuckey HSD post-hoc test). **(c)** Coefficient of variation of mean cystacanth volume within a host according to infection intensity. Numbers in parentheses or inside bars are sample sizes.

*Pomphorhynchus laevis* cystacanth volume was influenced by both parasite sibship and infection intensity, but not by host size nor by interactions between these factors (Global linear model: *F*_*9, 228*_ = 6.06, *P* <0.0001; Sibship effect: *F*_*7,228*_ = 2.83, *P* = 0.008; Parasite intensity effect: *F*_*2,228*_ = 20.81, *P* <0.0001). Cystacanths from two sibships differed significantly from other clutches (Figure 2a), and mean cystacanth volume per host decreased with infection intensity (Figure 2b). Interestingly, the volume of the largest cystacanth was not influenced by host size or intensity of infection, but only by parasite sibship (*F*_*7, 230*_ = 2.06, *P* = 0.047; Figure 2a, b). Both parasite sibships that showed significantly different sizes of the largest cystacanth also showed differences in mean cystacanth size (Figure 2a).

Only infection intensity influenced the variation coefficient of cystacanth volume within a host; host size, parasite sibship and interactions were not significant (Global model: *F*_1,156_ = 14.72, *P* = 0.0002). Parasite size was more variable in hosts with more than two cystacanths than in hosts with only two co-infecting *P. laevis* (Figure 2c). It is worth noting that this trend remains if the category “more than two cystacanths” is subdivided into more categories (results not shown).

### Host phototaxis

Overall, early phototaxis scores had a median of 1 (interquartiles: 0 – 3), and late phototaxis scores had a median of 6 (interquartiles: 2 – 9). For analyses, we therefore categorized our scores as “scores <1” and “scores ≥ 1” for early phototaxis, and “scores < 6” and “scores ≥ 6” for late phototaxis. Since infection intensity and cystacanth volume are two dependant variables, this relationship was analyzed separately according to infection intensity.

Early phototaxis scores were not influenced by parasite sibship or mean cystacanth volume (Global models: *χ²* = 1.38, *P* = 0.24; *χ²* = 1.54, *P* = 0.23, *χ²* = 0.02, *P* = 0.89 for one, two and more than two parasites per host, respectively).

**Figure 3:**
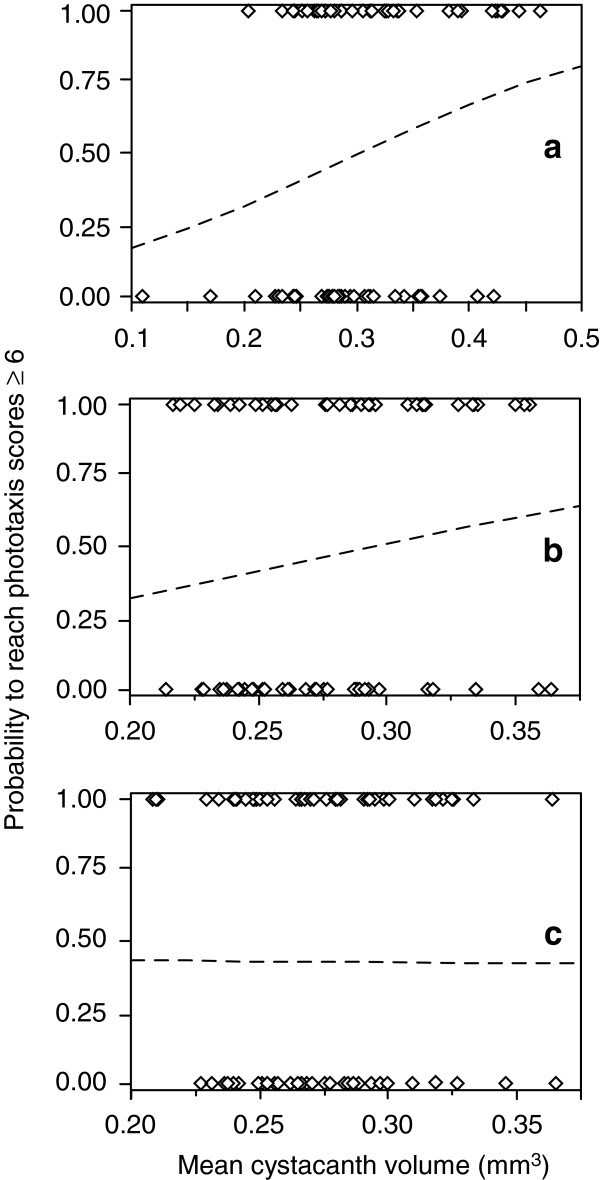
**Probability of reaching high phototaxis scores in gammarids (≥ 6) according to mean cystacanth volume. (a)** Hosts infected by one cystacanth. **(b)** Hosts harbouring two cystacanths. **(c)** Hosts infected by more than two cystacanths. Diamonds are real data, curves are those calculated by the logistic regression model.

In the analyses of late phototaxis scores, parasite sibship was never retained as a significant factor. Mean cystacanth volume had a weak but significant effect on late phototaxis in single infections, larger parasites having higher probabilities of expressing high phototaxis scores (*χ²* = 4.11, *P* = 0.04, Figure 3a). In hosts infected with two and more than two parasites, mean cystacanth volume did not influence late phototaxis (*χ²* = 1.48, *P* = 0.22 and *χ²* = 0.001, *P* = 0.97, respectively; Figure 3b, c). There was no relationship between the variation coefficient of cystacanth volume and host late phototaxis (*χ²* = 0.34, *P* = 0.56 and *χ²* = 0.15, *P* = 0.70 for hosts infected by two *P. laevis* and more than two parasites, respectively). Finally, analyses using the volume of the largest cystacanth within a host as a factor were similar to those using the mean cystacanth volume; largest cystacanth volume had no effect on early or late phototaxis in hosts harbouring two and three or more parasites (*χ²* = 1.65, *P* = 0.20 and *χ²* = 0.04, *P* = 0.85, respectively for early phototaxis scores; *χ²* = 1.60, *P* = 0.21 and *χ²* = 0.12, *P* = 0.73, respectively for late phototaxis).

## Discussion

Our study confirmed that *P. laevis* cystacanth size was influenced by intraspecific competition. This is consistent with previous studies on crowding effects on acanthocephalan size [8,14,32]. In particular, Cornet [32] found similar results when co-infecting *P. laevis* were unrelated (i.e. originated from different clutches). Interestingly, our results indicate that the size achieved by the largest cystacanth is always the same, regardless of the number of competitors. All cystacanths sharing a host may thus not equally suffer from competition. As a consequence, cystacanth size is more variable in hosts harbouring three or more cystacanths. Competition between cystacanths seemed to act on both cystacanth size variability and mean cystacanth size.

Since the largest cystacanth within a host reached a similar size in single and multiple infections, total parasite volume increased with the number of co-infecting cystacanths. Such correlation is believed to occur mostly among genetically unrelated parasites because related parasites should exploit a common host in a more cooperative way [35-37]. Here, all competing parasites came from the same clutch and were therefore *a priori* strongly related. We thus follow Keeney *et al.*[38] in their conclusion that genetic relatedness among co-infecting parasites is not inevitably the main factor acting on parasite growth. In addition, we found significant differences in *P. laevis* size between sibships. This difference was mainly due to two sibships where both the average and the largest cystacanth sizes were higher or lower than in other sibships. This variation suggests either genetic or maternal effects on larval size. Since each female parasite came from a different fish definitive host, effects of female *P. laevis* environment (i.e. definitive host) on offspring life-history traits are also possible.

In single infections, larger cystacanths had higher probabilities of inducing strong late phototaxis scores in their hosts. Positive relationships between host manipulation and parasite size have been found in few other host-parasite systems [25,39]. In these cases, it was difficult to discriminate between size and age of parasites as the main trigger of this relationship. Here, potential age effects are eliminated by our synchronized experimental procedure. Larger cystacanths should thus be advantaged in the absence of intra-host competition, assuming that higher phototaxis scores in amphipods increase *P. laevis* transmission probabilities to the final host. Franceschi *et al.*[23] previously found that early phototaxis is influenced by the rapidity of *P. laevis* development (fast-developing cystacanths altered less host behaviour than their slow-developing conspecifics). Both components of host behavioural modification (i.e. early and late phototaxis) are therefore modulated by two different parasite life-history traits. Early phototaxis, i.e. intensity of behavioural change induced by a parasite that has just reached the cystacanth stage, is influenced by parasite developmental rate. Late phototaxis, i.e. maximal intensity of behavioural change induced by an old cystacanth, is influenced by final larval size.

We found no effect of the number of co-infecting parasites on host behavioural manipulation intensity, a result that contrasts with those of Franceschi *et al.*[26]. However, this previous study used a mix of parasites from different clutches, and of different geographic origins, for their experimental infections. The difference between the two studies can therefore come from differences in the changes in the intensity of phototaxis scores among parasite populations [40]. If parasite number *per se* did not influence host behavioural alteration, the positive relationship between mean cystacanth size and host phototaxis was influenced by the number of parasites. This relationship was detected in single infections but disappeared in multiple infections. The absence of such a relationship under intra-host competition remains to be explained. Brown *et al.*[25] suggested that the largest of co-infecting parasites should have the strongest effect on host behavioural alterations. However, our data do not support this hypothesis. We found that the size of the largest *P. laevis* cystacanth did not decrease with infection intensity, indicating that the competition for size among co-infecting cystacanths affect all but this “winner” individual. Nevertheless, no effect of this “winner” parasite size on behavioural changes was found. Absence of relationship between parasite size and host manipulation in multiple infections could be due to parasites competing to reach high larval size not being able to invest in further behavioural manipulation, as parasites would do under no competition.

## Conclusion

By stressing parasite size effects on host manipulation, this study illustrates the complexity of the variability observed in parasite-induced host alterations, emphasizing behavioural manipulation as a highly condition-dependant phenomenon [41]. The need to account for all possible factors (parasite, host and/or environment-related) seems crucial to fully understand competition patterns between parasites and possible effects on transmission rates.

## Competing interests

The authors declare that they have no competing interest.

## Authors’ contributions

TR and LB conceived the study. LD and NF performed the experiment, collected the data and outlined the manuscript. LD, CL and TR realized the analyses. LD, TR and CL wrote the MS. All authors read and corrected earlier versions and approved the final manuscript.
